# A cerebral arteriovenous malformation mistakenly diagnosed as dry eye and glaucoma: a case report

**DOI:** 10.1186/s12886-019-1160-8

**Published:** 2019-07-12

**Authors:** Shuhao Shen, Xiaoyong Liu, Jian Chen, Chengyou Yang, Changzheng Shi, Qing Zhou

**Affiliations:** 1 The Department of Ophthalmology, The First Affiliated Hospital of Jinan University, No. 613, Huangpu Avenue, Tianhe District, Guangzhou, Guangdong Province China; 2 The Department of Neurosurgery, The First Affiliated Hospital of Jinan University, Guangzhou, Guangdong Province China; 3 Medical Imaging Division of the First Affiliated Hospital of Jinan University, Guangzhou, Guangdong Province China

**Keywords:** cAVM, DSA, Hyperaemia, Glaucoma

## Abstract

**Background:**

To report the first case of a cerebral arteriovenous malformation (AVM) with ocular symptoms and review the characteristics of this case and the main point of confusion for the diagnosis of such a case.

**Case presentation:**

A 58-year-old woman presented to the ophthalmology clinic with 1 and a half years of right eye redness, ocular hypertension and recurrent headache. One and a half years ago she was diagnosed with right eye dry eye and glaucoma and had received treatment according to this diagnosis. However, none of the treatments led to any improvement in redness and headache. Physical examination revealed dry eye and severe corkscrew hyperaemia with dilated vessels in the right eye. The results of fundoscopic examination of both eyes were normal. After we considered that the symptoms may be related to abnormal intracranial vessels, computed tomography angiography and venography (CTA + CTV) were performed, and the results showed an arteriovenous malformation in the right parietal-occipital area in the brain. The AVM was definitively located by further examination with digital subtraction angiography (DSA). After AVM endovascular embolism treatment, the conjunctival congestion of the right eye was significantly relieved, and the intraocular pressure decreased to normal.

**Conclusion:**

In clinical practice, when corkscrew hyperaemia accompanied by neurological symptoms is found, cerebral vascular diseases might be considered. In this case, the ophthalmologist’s diagnosis should combine disease history and imaging examination.

## Background

A cerebral arteriovenous malformation (cAVM) is a cluster of vascular masses developed from congenital cerebrovascular dysplasia with dysregulated differentiation in the early stage of embryonic development. The acquired factors that lead to cerebral arteriovenous malformations include specific diseases or external interventions, and these factors could cause the malfunction of cerebral angiogenesis or change the normal physiological structures of cerebral vessels. The cAVM symptom occurrence rate of intracranial haemorrhage, headache, and dizziness were 43.4, 17.3 and 24.9%, respectively, of which headache was the second leading symptom [1]. Furthermore, differences in the location of the cAVM can cause symptoms in the associated organs. Omer Faith Nas [2] reported one case in which a patient with a space-occupying lesion compatible with a pial AVM in the right occipital region 1 year prior had symptoms of blurred vision, dizziness, nausea, and headache localized to the occipital region. This case reminds us that intracranial vascular lesions can also cause related ophthalmic symptoms.

The superior ophthalmic vein collects the blood from the anterior ciliary vein, venae vorticosae, and vena centralis retinae. Due to the AVM being directly or indirectly connected to the superior ophthalmic vein, the high pressure of the superior ophthalmic vein prevents the aqueous fluid and blood of the sclera and conjunctiva from flowing backward. Furthermore, it will lead to severe conjunctival injection symptoms, such as increased intraocular tension and enlarged and tortuous veins of the conjunctiva and sclera.

## Case presentation

**Fig. 1:**
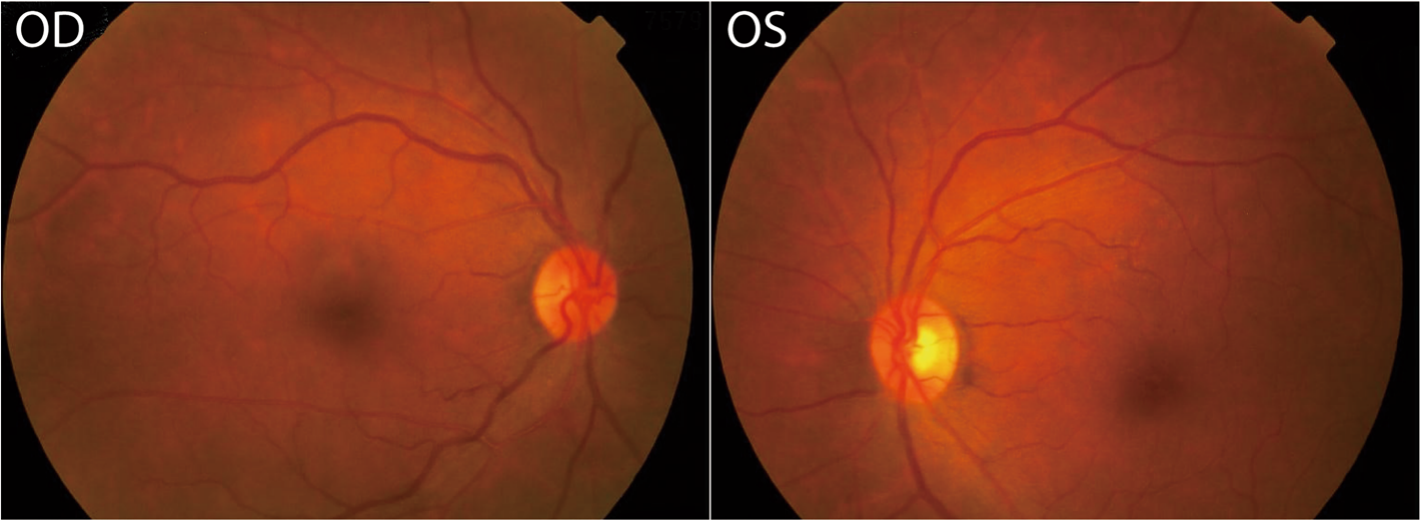
The fundoscopic images of both eyes before treatment. No obvious lesions were found in the optic disc and fundus vessels of either eye

1A 58-year-old woman presented to the ophthalmology clinic with 1 and a half years of right eye redness and ocular hypertension. Tracing her history, she had recurrent headaches for several years without a history of head trauma. She was diagnosed with right eye dry eye and glaucoma and received treatment including an NSAID, immunosuppressive therapy, a prostaglandin analogue and β-blocker combination medication for eye pressure reduction, and even embolization of the lacrimal punctum. However, all the treatments only slightly decreased the intraocular pressure with no improvement in eye redness and headache. On examination, the visual acuity and intraocular pressure in the right eye of the patient were 20/40 and 20 mmHg after the aforementioned treatment, while these variables in the other eye were 20/25 and 14 mmHg, respectively. Physical examination revealed exophthalmos and severe corkscrew hyperaemia with dilated vessels in the right eye. The results of fundoscopic examination of both eyes were normal. No obvious lesions were found in the optic disc and fundus vessels of either eye (Fig. 1).

**Fig. 2:**
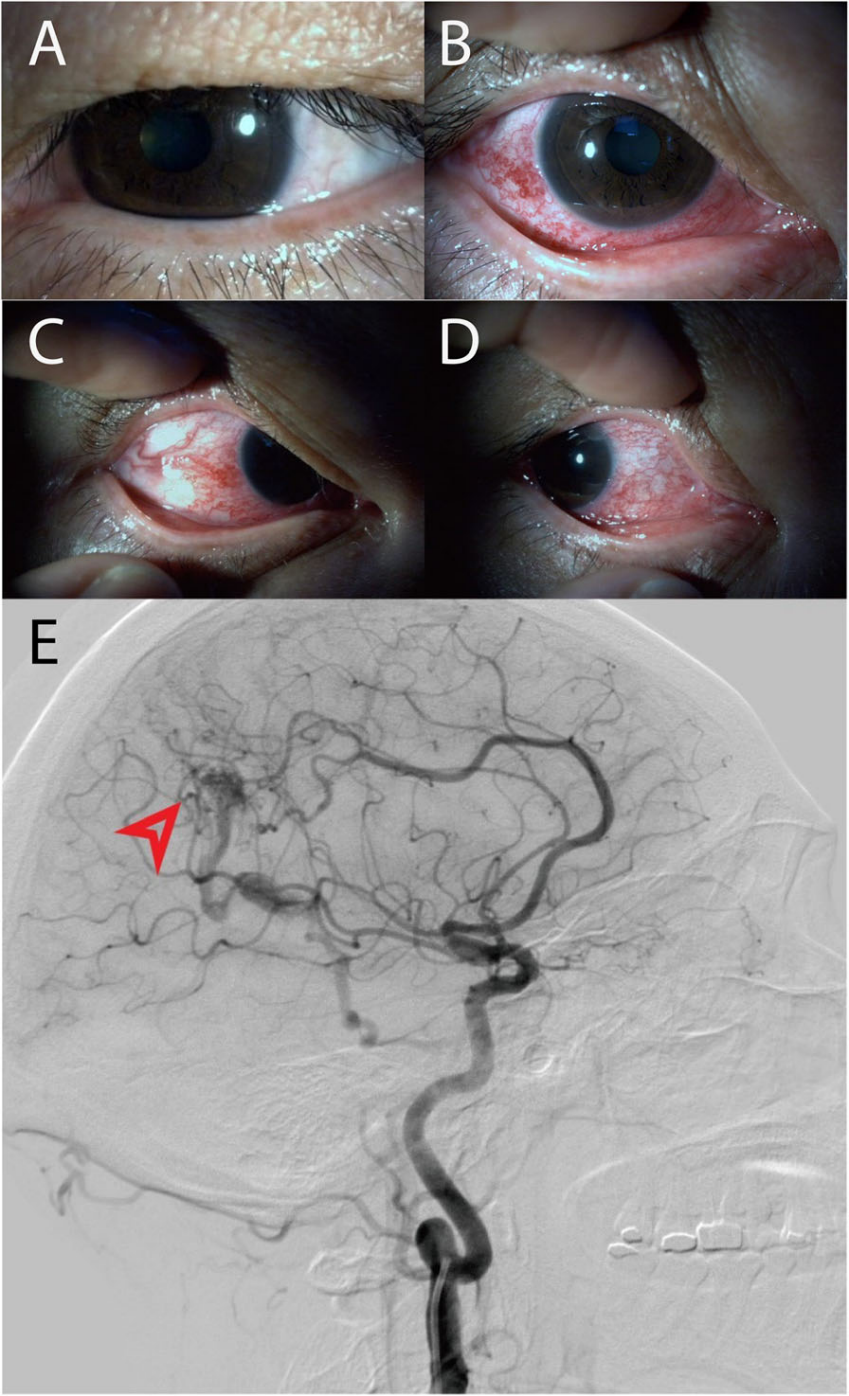
The appearance of the both eye There was no obvious hyperaemia in the left eyes (**a**). The right eye exhibits severe diffuse corkscrew hyperaemia (**b-d**). Imaging revealed that the AVM (The red arrow) is fed by the posterior cerebral artery and pericallosal artery and drains to the medial atrial vein (**e**)

After we considered that the symptoms may be related to abnormal intracranial vessels, computed tomography angiography and venography (CTA + CTV) were performed, and the results showed an arteriovenous malformation (AVM) in the right parietal-occipital area in the brain. The AVM was definitively located by further examination with digital subtraction angiography (DSA). DSA revealed that the AVM is fed by the posterior cerebral artery and pericallosal artery and is drained by the medial atrial vein. Then, this malformed venous structure could lead the arterial blood flowing into the superior ophthalmic vein (Fig. 2).

With these results, the patient was ultimately diagnosed with an “occipital lobe cerebral arteriovenous malformation”, and surgical treatment was given.

**Fig. 3:**
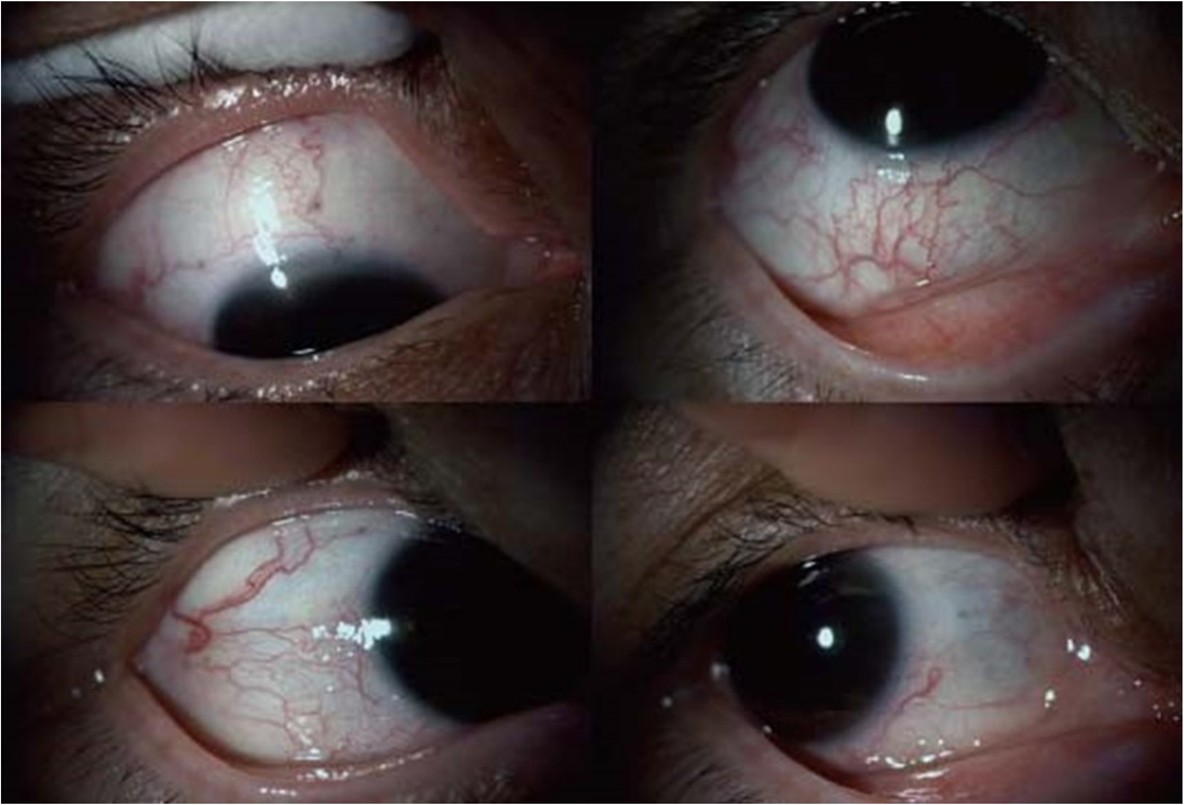
The appearance of the right eye after endovascular embolism The conjunctival congestion of the right eye was significantly relieved

After the general cerebral angiography probe reached the right occipital lobe AVM embolism, the arteriovenous malformation mass was blocked. This block led to restoration of normal blood flow of the superior ophthalmic vein, in which the conjunctival congestion of the right eye was significantly relieved (Fig. 3) and the intraocular pressure decreased to normal (14–15 mmHg).

## Discussion and conclusion

### Aetiology and classification

Cerebral arterial malformations are congenital vascular anomalies. They can present with bleeding and compression effects on surrounding tissues [2]. The authors believe that AVMs will gradually cause different degrees of symptoms with age and deterioration of vascular conditions. Due to the absence of capillaries between cerebral veins and cerebral arteries in the area of cerebrovascular diseases, arteriovenous communication and vascular regulation mechanism barriers are formed, leading to cerebral blood vessel flow turbulence. In this case, due to the presence of an arteriovenous malformation of the right parietal-occipital lobe, the arterial blood flow passed through local internal atrial veins via the superior petrosal sinus, flowed back to the jugular veins, and then flowed backward to the superior ophthalmic vein, causing the high pressure in the superior ophthalmic vein. In this case, early CTA + CTV examination revealed that the right superior ophthalmic veins were enlarged and tortuous, which was in accordance with such changes. Since the drainage vessels on both sides were not connected, the affected eye of the patient was on the same side as the malformed mass, and only one side was affected.

### Clinical presentation and differential diagnosis

The main clinical features of a cAVM include intracranial haemorrhage, headache, dizziness and convulsion, as well as neurological dysfunction, brain tissue swelling, etc. Because the patient did not have any history of obvious craniocerebral trauma, the eye disease was not considered to have been caused by intracranial vascular lesions. Hence, relative imaging examinations were not conducted. Therefore, the patient was once mistakenly diagnosed as “1. Dry eye in the right eye; 2. Glaucoma in the right eye”. First, a cAVM and dry eye can be differentiated by corkscrew hyperaemia, and the dry eye would not cause recurrent headache. Second, a cAVM can result in increased intraocular pressure, orbital pain, and headache but without typical keratic precipitates (KP) and iridociliary disorder, which distinguish this condition from secondary glaucoma. Furthermore, the common anti-glaucoma therapy was not effective for this case. Conjunctival congestion, which is usually characterized by dilation of blood vessels away from the limbus of the cornea, also occurs frequently in various types of conjunctivitis, and conjunctivitis is not accompanied by increased intraocular pressure.

After several ineffective treatments, we considered intracranial vascular diseases, such as a carotid-cavernous fistula (CCF). A CCF refers to an aberrant connection between the internal carotid artery (ICA), the external carotid artery (ECA) or any of their branches within the cavernous sinus [3]. The symptoms and signs of a CCF always include eyelid swelling, proptosis, chemosis, and corkscrew hyperaemia, which is similar to this case. At present, the diagnosis of a cAVM and CCF mainly depends on digital subtraction angiography (DSA), which is considered to be the gold standard for the diagnosis of a cerebral artery malformation and can be used to identify a CCF and an AVM. However, since it is an invasive and costly examination, it is not appropriate as a requirement for the purpose of early diagnosis. CTA + CTV can clearly show the 3D structure of the malformed mass, as well as locate the position of the lesions precisely. In addition, the conditions of cranial arteries and veins, as well as the direction of flow of draining veins, can be shown clearly [4]. The mature application of combined techniques including CTA, CTV and MRI have provided visualized 3D images of the relation between lesions and their surrounding structures for clinicians, which is more suitable for early diagnosis.

Many patients only present clinically with neurological symptoms. Therefore, when ophthalmic symptoms occur, ophthalmologists would not consider intracranial disease. In clinical practice, when corkscrew hyperaemia accompanied by neurological symptoms is found, cerebral vascular diseases might be considered. In this case, the ophthalmologist’s diagnosis should combine disease history and imaging examination.

In conclusion, it is difficult for an ophthalmologist to diagnose a cAVM without any imaging examination; careful examination of the medical history is a necessary part of diagnosing this disease. According to the medical history, the ophthalmologist should conduct a radiographic examination to decide between conservative treatment or embolization surgery to delay disease development of a cAVM and prevent severe complications, such as intracranial haemorrhage. Additionally, a cAVM can result in ocular symptoms, which is a very unusual situation but to which it is necessary for the ophthalmologist to pay attention.

### Treatment

At present, the treatment methods for arteriovenous malformation mainly include excision by microsurgery, endovascular embolism, and stereotactic radiotherapy. Endovascular embolization has been playing an increasingly important role in AVM therapy due to its convenience and minimally invasive features [5]. Due to its lower invasiveness with less damage to the brain, the interventional embolization via vessels used in this case has become the main treatment method for arteriovenous malformation [6].

## Data Availability

All data have been presented within the manuscript and in the form of images.

## Abbreviations

cAVM: Cerebral arteriovenous malformation
CCF: Carotid-cavernous fistula
CTA: Computed tomography angiography
CTV: Computed tomography venography
DSA: Digital subtraction angiography
ECA: External carotid artery
ICA: Internal carotid artery
KP: Keratic precipitates
MRI: Magnetic resonance imaging
